# Management of pharmaceutical ICH M7 (Q)SAR predictions – The impact of model updates

**DOI:** 10.1016/j.yrtph.2020.104807

**Published:** 2020-10-13

**Authors:** Catrin Hasselgren, Joel Bercu, Alex Cayley, Kevin Cross, Susanne Glowienke, Naomi Kruhlak, Wolfgang Muster, John Nicolette, M. Vijayaraj Reddy, Roustem Saiakhov, Krista Dobo

**Affiliations:** a Genentech, Inc., 1 DNA Way, South San Francisco, CA, 94080, USA; b Gilead Sciences, 333 Lakeside Drive, Foster City, CA, USA; c Lhasa Limited, Granary Wharf House, 2 Canal Wharf, Leeds, UK; d Leadscope, Inc. an Instem Company, 1393 Dublin Rd, Columbus, OH, 43215, USA; e Novartis Pharma AG, Pre-Clinical Safety, Werk Klybeck, CH-4057, Basel, Switzerland; f FDA Center for Drug Evaluation and Research, Silver Spring, MD, 20993, USA; g Roche Pharmaceutical Research & Early Development, Pharmaceutical Sciences, Roche Innovation Center Basel, F. Hoffmann-La Roche Ltd, Grenzacherstrasse 124, 4070, Basel, Switzerland; h AbbVie Inc., Pre-clinical Safety, 1 North Waukegan Road, North Chicago, IL, USA; i Merck & Co., Inc., West Point, PA, 19486, USA; j Multicase Inc., 23811 Chagrin Boulevard, Suite 305, Beachwood, OH, 44122, USA; k Pfizer Worldwide Research & Development, 558 Eastern Point Road, Groton, CT, 06340, USA

**Keywords:** Q)SAR, Mutagenic, Impurities, ICH M7, Computational models, Version update, Pharmaceuticals

## Abstract

Pharmaceutical applicants conduct (Q)SAR assessments on identified and theoretical impurities to predict their mutagenic potential. Two complementary models—one rule-based and one statistical-based—are used, followed by expert review. (Q)SAR models are continuously updated to improve predictions, with new versions typically released on a yearly basis. Numerous releases of (Q)SAR models will occur during the typical 6–7 years of drug development until new drug registration. Therefore, it is important to understand the impact of model updates on impurity mutagenicity predictions over time. Compounds representative of pharmaceutical impurities were analyzed with three rule- and three statistical-based models covering a 4–8 year period, with the individual time frame being dependent on when the individual models were initially made available. The largest changes in the combined outcome of two complementary models were from positive or equivocal to negative and from negative to equivocal. Importantly, the cumulative change of negative to positive predictions was small in all models (<5%) and was further reduced when complementary models were combined in a consensus fashion. We conclude that model updates of the type evaluated in this manuscript would not necessarily require re-running a (Q)SAR prediction unless there is a specific need. However, original (Q)SAR predictions should be evaluated when finalizing the commercial route of synthesis for marketing authorization.

## 1. Introduction

Impurities in pharmaceuticals are unavoidable. The synthesis of a chemical drug substance involves starting materials, intermediates, reagents, by-products, etc. that have the potential to be present in the drug product as impurities. In addition, drug substances and drug products can also degrade, which could further result in the presence of unintended chemicals. The ICH Q3A (R2) (ICH, 2006a), and ICH Q3B (R2) (ICH, 2006b) guidelines were developed in order to minimize exposure to impurities and ensure patient safety. ICH M7 (R1) (ICH, 2017) was published in order to control for a potentially toxic subset of impurities, i.e., DNA reactive, mutagenic impurities. The guideline describes the process for identification, categorization, qualification, and control of impurities to limit carcinogenic risk. It is important to identify mutagenic impurities as they are controlled to lower limits than non-mutagenic compounds. The limit for a mutagenic impurity without adequate corresponding *in vivo* carcinogenicity data is based on the threshold of toxicological concern (TTC). The TTC derived for mutagenic compounds was determined by analyzing the carcinogenic potency of known carcinogens and deriving the exposure that has a theoretical excess cancer risk of 1 in 100,000 over background (Kroes et al., 2004; Muller et al., 2006). The resulting TTC based on this analysis is 1.5 μg/day for chronic (administration for >10 years) exposure.

(Quantitative) Structure Activity Relationship ((Q)SAR) predictions are an important part of hazard characterization in the context of ICH M7 (R1) application. (Q)SAR models make predictions of biological activity based on chemical structure and, consequently, only a chemical structure instead of any physical material for experimental testing is needed for a known or potential impurity to generate a prediction of its mutagenicity. Specifically, models used for ICH M7 purposes predict the outcome of the bacterial reverse mutation assay (i.e., Ames assay (Ames et al., 1973a; Ames et al., 1973b; Gee et al., 1994)), which is the recommended experimental assay to identify DNA reactive compounds. (Q) SAR models were used in this context even prior to the adoption of ICH M7 (Dobo et al., 2006). Due to the large number of impurities needing evaluation during the development of a new drug, (Q)SAR models offer an attractive alternative to empirical testing for each impurity, which itself may require the time-consuming process of synthesis and purification of the impurity to conduct the assay. (Q)SAR models can provide the high-throughput capacity needed to generate predictions for hundreds of impurities in a fraction of the time, with predictions achieving the high sensitivity and negative predictivity needed to protect patient safety. If an impurity is predicted as mutagenic, one can then choose to demonstrate control of the impurity at or below the TTC or choose to do follow-up mutagenicity testing in the bacterial reverse mutation assay in accordance with OECD 471 and ICH S2 (R1) guidelines.

ICH M7 (R1) recommends that two complementary (Q)SAR models are used, one expert rule-based and the other a statistical-based model. Expert rule-based models generate predictions based on knowledge of structure-activity relationship patterns derived by experts, who extract structural alerts and mitigating features from datasets and the published literature and encode the patterns in the software (Barber et al., 2017). Statistical-based models are agnostic of external rules and are instead trained by an algorithm that extracts information from a training set without the inclusion of prior knowledge. Both types of (Q)SAR models (i.e., rule- or statistical-based) should meet the general validation principles set forth by the Organization for Economic Co-operation and Development (OECD) (OECD, 2007). Furthermore, the ICH M7 (R1) guideline states that in the absence of structural alerts from two complementary methodologies, it can be concluded that an impurity is non-mutagenic. If warranted, expert knowledge can be used to overrule any prediction, positive, negative or equivocal. Expert knowledge has been described in other publications and requires a description with supporting information as to why the prediction is overruled (Barber et al., 2015; Powley, 2015; Amberg et al., 2016). Expert review has been shown to be an important process to enhance the reliability of (Q)SAR predictions (Dobo et al., 2012; Sutter et al., 2013).

The process of identifying impurities and assessing their mutagenic potential is the responsibility of the pharmaceutical applicant and is typically done in-house for larger organizations, and by consultants for smaller organizations. The determination of which impurities should undergo (Q)SAR varies between different organizations. The workflow in an industry setting may be as simple as the process chemist(s) deciding and assembling the list, or more of a cross-functional collaboration, involving a joint review by an internal group of experts to determine which structures are relevant to assess.

Actual and potential impurities as well as degradation products, as defined by ICH M7 (R1), require (Q)SAR assessment, if there are no mutagenicity data available. Some organizations may however choose to review the entire synthetic scheme. The process of impurity assessment is not static; during the course of development, the understanding of the synthetic route and, thus, associated impurities, continues to evolve. The synthetic route may be changed for various reasons such as scalability, minimizing environmental waste, process safety, economic reasons, etc. The formulation of the drug product also continues to evolve to maximize stability and systemic absorption of the drug substance. All of these changes can result in new impurities that need to be computationally evaluated. (Q)SAR models are also not static over time. Data and knowledge used to build the models evolve and grow continuously, requiring software developers to update their statistical models or add/modify rules in their rule-based systems or any associated reference sets. Additionally, feedback from software users on model strengths and weaknesses, identified through their routine use, is sent back to model developers to focus their efforts on targeted upgrades for future versions. This includes changes to the structure-activity relationships encoded in the models along with internal data made available to the model developers. Modifications to a (Q)SAR model are delivered to pharmaceutical applicants as version updates and this is commonly done on an annual basis. Changes to the models are described in the technical release notes, which are provided to pharmaceutical applicants for their awareness. However, the subtle effects that upgrades can have on specific model predictions are not always known. Whilst changes to (Q)SAR models are necessary to capture new information or new data, despite being ultimately beneficial, they can be difficult for pharmaceutical applicants to manage. Drug development is a slow process and 6–7 years can transpire from the time of an investigational new drug (IND) application to the first marketing application (FDA, 2019). Updating the (Q)SAR predictions on a yearly basis before the market application is generally impractical for many pharmaceutical applicants, given the number of impurities that need to be evaluated for each program in development. The ICH M7 guidance does not provide any recommendations regarding the management of software updates in the context of contemporizing (Q)SAR predictions. Consequently, applicants have developed different practices. Some may update (Q)SAR predictions every time a new software version is released, others may not reevaluate between IND and first marketing application, and still others may conduct periodic updates at specific points in their development process, such as when manufacturing new batches, for example. In the recently released ICH M7 (R2) Q&A document (ICH, 2020), the lack of guidance has been addressed and it is stated that re-running (Q) SAR predictions during development is not necessary, although it is recommended in preparation for marketing. This guidance was however not available at the time this study was initiated.

The main objectives of this study were to understand the magnitude and significance of changes to (Q)SAR predictions over time, and based on that understanding, recommend a best practice for updating (Q)SAR predictions during drug development. For this purpose, we assembled a random dataset that was then assessed in tools from three different commercial software vendors (Lhasa Limited (Lhasa, 2020), Leadscope Inc. (Leadscope, 2020) and MultiCASE Inc. (MultiCASE, 2020)), covering models released over a time period of 4–8 years. The analysis presented here was based on thousands of compounds covering a chemical space modeled to represent pharmaceutical impurities, since this was considered most representative of the practical situation at hand. Furthermore, the compounds selected for analysis had no experimental bacterial reverse mutation results available as the objective of the investigation was only to assess changes in (Q)SAR predictions over time, rather than assess the accuracy of predictions over time. The results of this analysis form a good basis for providing scientific recommendations for pharmaceutical applicants on when it may be appropriate to update the original predictions using more contemporary software versions.

## 2. Data and methods

### 2.1. Creating a public dataset relevant to ICH M7 chemical space for evaluating changes to in silico predictions

A test set was created to represent the chemical space and compound class distribution commonly encountered for drug impurities evaluated in an ICH M7 use case. Compounds were extracted from the PubChem database (NIH-NLM, 2019) by matching them to structurally similar compounds in the Lhasa Vitic Intermediates database. The Vitic Intermediates database project is a data sharing initiative organized by Lhasa Limited in which proprietary mutagenicity data for drug intermediates, impurities and synthetic reagents are shared within a consortium of pharmaceutical companies for the benefit of all members (Elder et al., 2015; Vitic, 2020). The 2017.1.0 Vitic Intermediates database contains 1341 proprietary impurity structures and the chemical space “populated” by the Vitic Intermediate dataset is probably the most relevant available for this evaluation. However, it should be noted as a caveat that this set represents a subset of chemicals that companies have chosen to share and does not necessarily cover the entire chemical space of drug development. Since the data themselves cannot be used directly, matching them to structurally similar compounds in the public domain provided a suitable alternative.

**Fig. 1.**
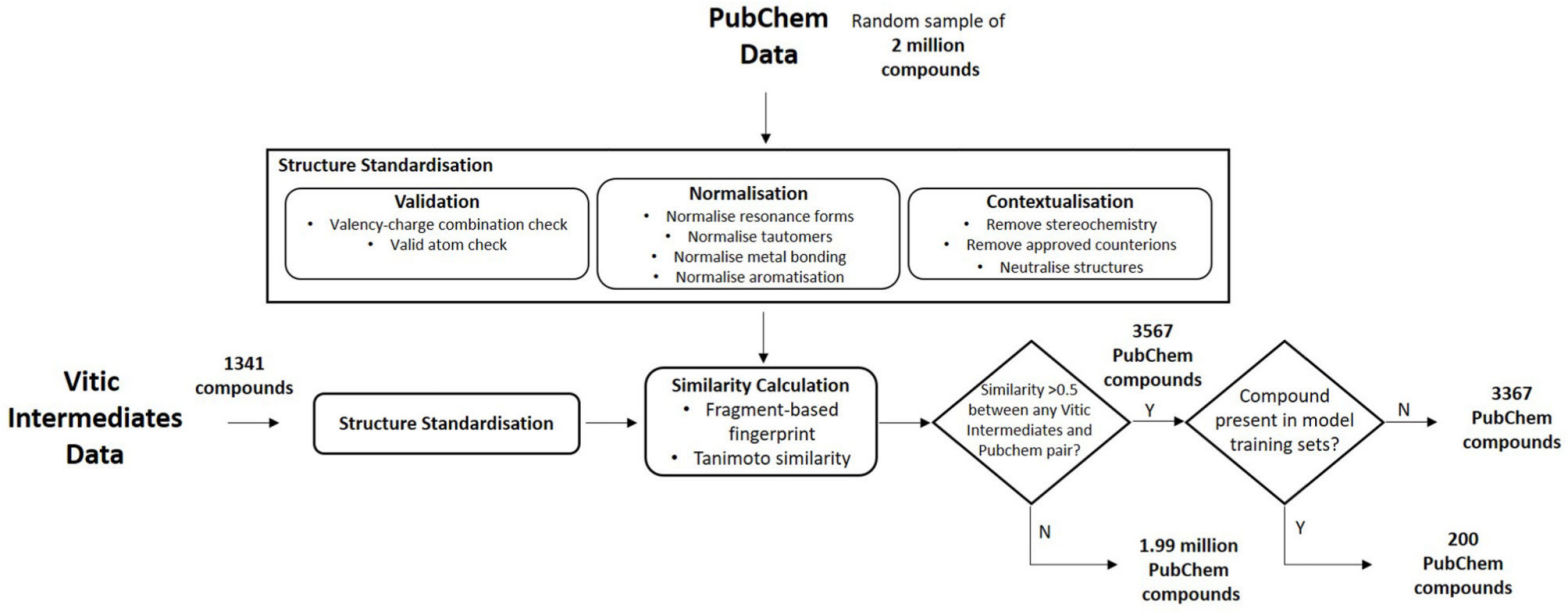
An illustration of the workflow used to generate a dataset of 3367 compounds from PubChem representing an area of chemical space and compound distribution relevant to the ICH M7 workflow.

Initially, a random sample of two million compounds was obtained from PubChem. Structures were then standardized by carrying out validation (checking for integrity of the chemical structure), normalization (ensuring that the structures are represented in a consistent manner) and contextualization (removing structural information which is not relevant in the current context, such as stereochemistry). Mixtures and compounds containing metals other than group I and II were removed from the dataset as part of this process. Structural similarity analysis was carried out between the PubChem compounds and standardized structures present in the Vitic Intermediates database in order to identify structures most representative of the chemical space contained in the proprietary database. The approach used the fragment-based fingerprinting method described by Hanser et al. (2014) to create fragment-based bit sets for each compound which were then compared using the Tanimoto similarity measure (Rogers and Tanimoto, 1960; Willett et al., 1998). A cut-off of 0.5 was used to select compounds from the PubChem dataset that were deemed sufficiently similar to those present in the Vitic Intermediate dataset. This produced an initial dataset of 3567 compounds. This dataset was further refined by removing any structures that were present in the training set of one or more (Q)SAR models used in this investigation, or in databases where experimental bacterial reverse mutation data were available. This resulted in a final dataset of 3367 compounds from PubChem (available in supplementary information), representing an area of chemical space presumably most relevant to drug intermediates, impurities and synthetic reagents and thereby relevant to the ICH M7 use-case (Fig. 1).

**Fig. 2.**
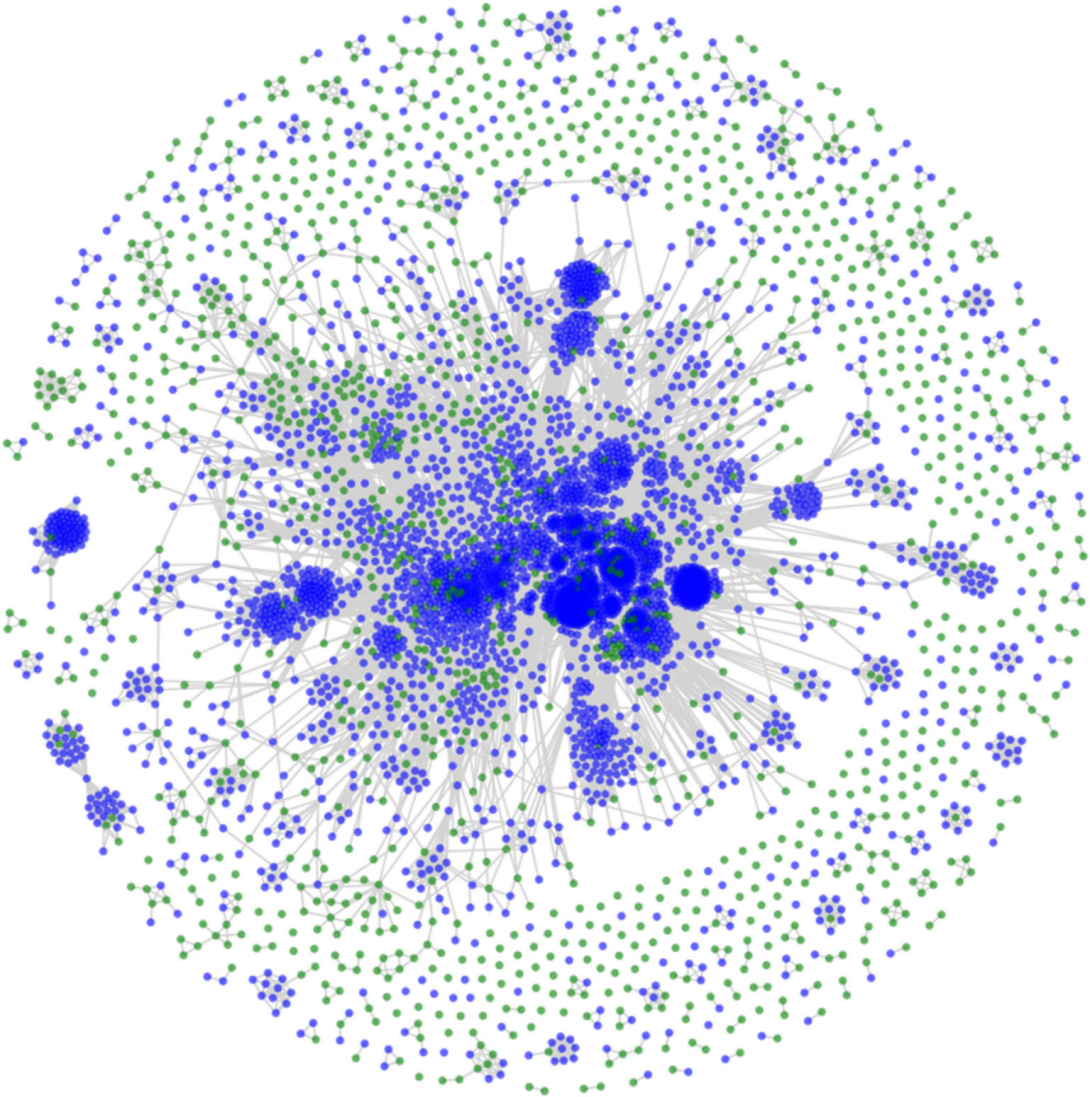
A similarity graph displayed with a force directed layout representing the chemical space represented by the Vitic Intermediates dataset (data points in green) when compared to the PubChem ICH M7 dataset (data points in blue).

To analyze the chemical space and distribution of chemicals covered by the PubChem sampled dataset and compare it to the chemical space covered by the Vitic Intermediates dataset, a similarity graph was generated using the same fragment-based fingerprinting method as earlier. The similarity graph was visualized using a force directed layout (Fig. 2). This analysis showed that the data points in both datasets are generally clustered around specific areas of chemical space in the diagram center. However, a significant minority of these points have few or no connections to other points in the datasets and are represented by small clusters or ‘singletons’ on the outside of the circle. The PubChem chemicals (represented by the blue dots in Fig. 2) generally cover the chemical space represented by the Vitic Intermediates dataset, as illustrated by their data points being associated with one or more Vitic Intermediates data points. The Vitic Intermediates dataset does contain some unique chemical space, when compared to that of PubChem. Since pharmaceutical compounds are, by design, in non-patented chemical space, there may be unique chemical features not found in publicly available data. Further analysis reveals that some Vitic Intermediate compounds have unique substitution patterns, even when their functional groups are represented in the PubChem dataset. The employed similarity metric may not see these compounds as being similar overall. However, since the (Q)SAR methods use fragment-based descriptors, this will not necessarily have implications in the coverage of the models. This is discussed further in the results section pertaining to out-of-domain results.

**Fig. 3.**
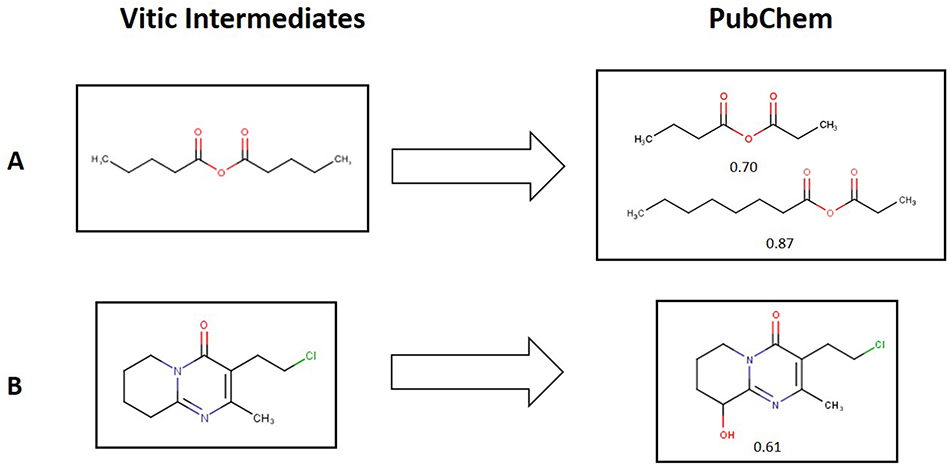
Similarity of some selected examples from the Vitic Intermediates dataset with the structures extracted from the PubChem database.

Examples of the typical relationships between the specific compounds present in the Vitic Intermediate dataset and the similar compounds present in PubChem are shown in Fig. 3. Each Vitic Intermediates compound was usually associated with several PubChem chemicals (Example A) but in some cases, one Vitic Intermediates compound was associated with only one PubChem compounds (Example B). There were also rare instances where the same PubChem compound was associated with multiple Vitic Intermediates compounds.

**Fig. 4.**
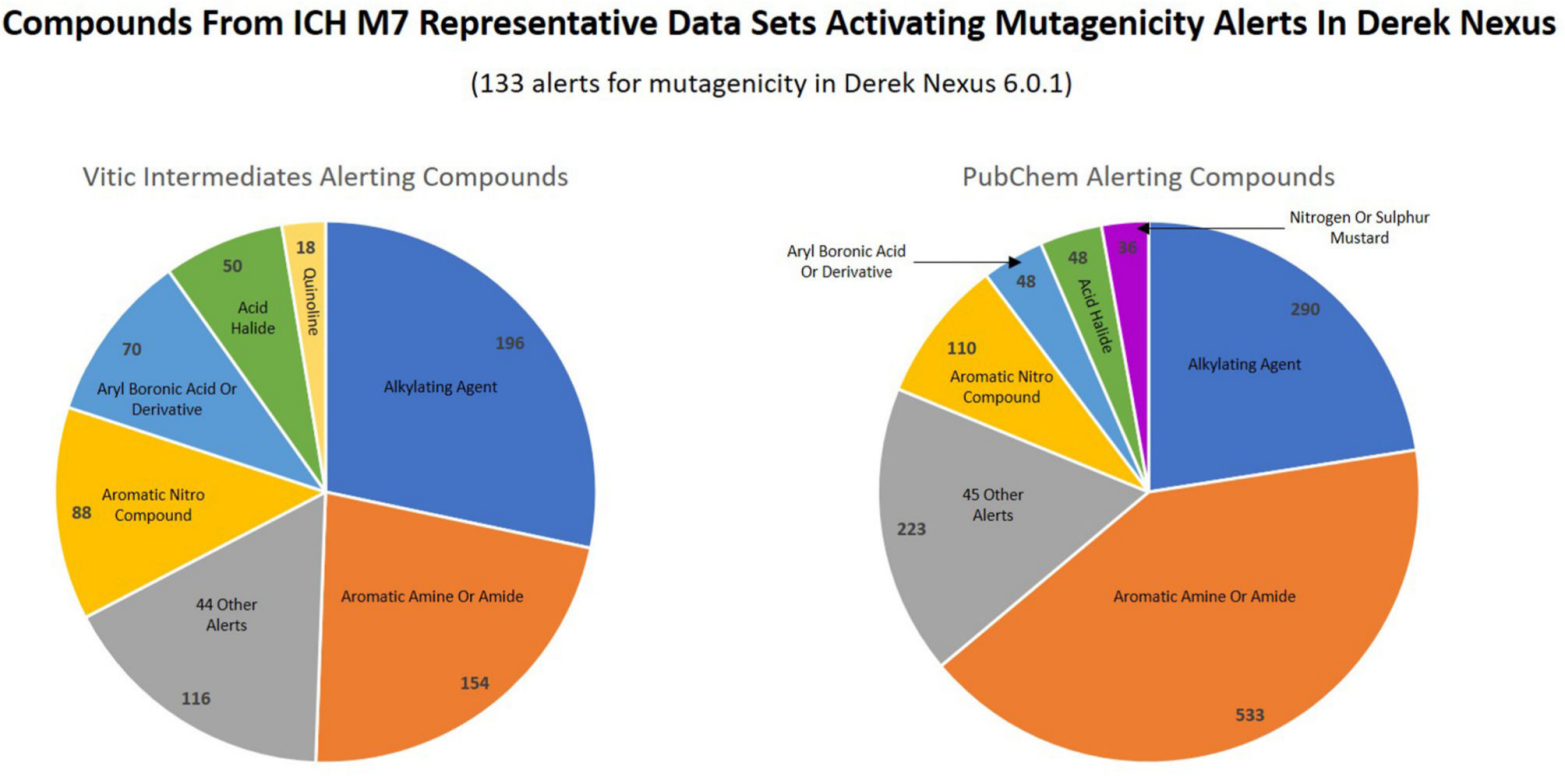
Pie charts illustrating the distribution of mutagenicity alerts activated in Derek Nexus version 6.0.1 by the Vitic Intermediates and PubChem datasets.

In order to investigate the ‘toxicophore space’ covered by both datasets, the chemicals were matched using the mutagenicity alerts present in the expert rule-based prediction system Derek Nexus. The frequencies of the different activated alerts for each dataset are shown in Fig. 4. Both datasets predominantly activated a small number (n = 6) of the same alerts in the knowledgebase, with the remainder (44 total alerts by the Vitic Intermediates compounds and 45 alerts by the PubChem compounds) of activated alerts falling into a mixed category of ‘other’. The two datasets also both activated the same alerts in the same relative proportions, indicating that the two datasets occupy a similar ‘toxicophore space’. There is a total of 133 alerts for bacterial mutagenicity in the Derek Nexus version 6.0.1 knowledge base and each dataset activated less than half of these alerts. These results are expected, as some alerting features are rarely encountered in drug impurities due to their limited use in drug synthesis. Additionally, some alerts may be very specific in their definition and only represented by a very small set of chemicals. Others may be based on a large number of chemicals and have a much broader scope. The toxicophores represented in our datasets reflect this, predominantly activating alerts more general in nature and less specific in their structural definitions (Fig. 4). It is clear from this analysis that the ‘toxicophore space’ covered by these datasets (and by extrapolation, those alerts important for the ICH M7 use case) does not necessarily reflect the total knowledge of toxicophores in bacterial mutation prediction systems, but rather a specific section of this knowledge. In that respect, this may be considered a limitation of this analysis, but since our focus is primarily on pharmaceutical impurities, it was not viewed as a concern for this particular study. In a separate comparison, alkylating agents (25.9%), aromatic amines/amides/N-hydroxylamines (20.8%), aromatic nitro derivatives (14.6%), acid chloride derivatives (5.8%), and alkyl aldehydes (4.7%) were the top five alerting features found in synthetic routes (Galloway et al., 2013). Therefore, while the toxicophore space was restricted, it was representative of the alerting features typically found as pharmaceutical impurities.

### 2.2. Computational models

The final PubChem test set was evaluated using current and historical versions of both statistical and rule-based models from Leadscope (LS), MultiCASE (MC) and Lhasa (LH). Details of the models used, as well as brief descriptions of major model revisions, are listed in the sections below and Tables 1–3. In general, changes to all models and alerts are reflective of the growing body of Ames test study data, changes in criteria for concluding that an Ames test result is positive (Williams et al., 2019) or negative (Landry et al., 2019), as well as better knowledge of the structure-activity relationships resulting in bacterial mutation for new classes of chemistry.

#### 2.2.1. LS models

The Leadscope Genetox Expert Alerts system has been updated yearly since its introduction in 2014 to include knowledge shared from corporate pharmaceutical applicants over the previous year through the Leadscope knowledge-sharing program. This program allows examination of proprietary corporate information under confidentiality restrictions through the use of Leadscope structure-activity fingerprints, designed to elucidate SARs for specific compound classes and thereby improve alert accuracy. This methodology was first introduced in the Genetox Expert Alerts version 3 (2016) and has been described in Ahlberg et al. (2016). Since 2014, the predictivity of several classes of compounds has been improved using this technique, and the results published for aromatic amines (Ahlberg et al., 2016) and aromatic N-oxides (Amberg et al., 2019b). Additional alerts updated during this 2014–2018 investigation period include: carboxylic and sulfonic acid halides, methyl halides, alkyl methyl halides, aromatic and aliphatic nitro groups, tri-substituted halogens, peroxides, quinolines, nitrones, alkyl esters, halo-amines, aromatic amides, hydrazines, polycyclic aromatics, fluorenes, and aryl boronic acids. The majority of these functionalities are present in the dataset used for this analysis and each year’s improvement to the alert set has potentially resulted in changes to the predictions for these classes of compounds.

**Fig. 5.**
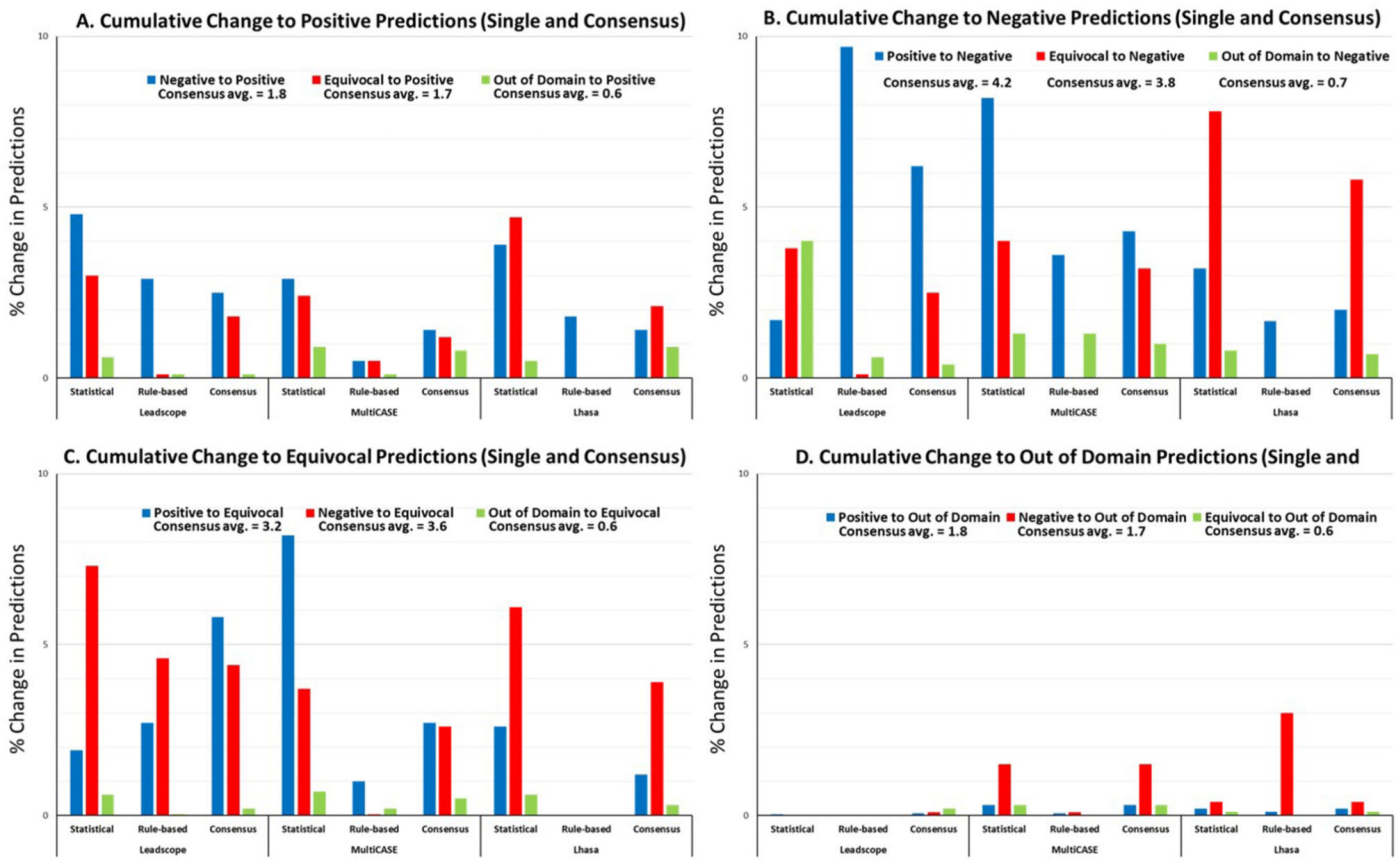
A–D Cumulative changes for each prediction category from 2014 to 2018. The numbers represent the average change.

Improvements in performance of the Leadscope Salmonella mutagenicity statistical model during the course of this investigation were primarily due to a substantial increase in the size of the training set (Landry et al., 2019) and incorporation of larger, more relevant chemical features (Stavitskaya, 2013). A new composite bacterial mutation statistical model was created in 2018 from a training set of 9189 compounds extracted from the Leadscope genetox database using an overall bacterial mutation prediction output The larger number of observations allowed construction of larger, more discriminating chemical structural features (i.e. more highly weighted features in the statistical model). Additionally, these features were made more chemically relevant through a software update ensuring inclusion of complete organic chemistry functional groups in a feature resulting in better sensitivity (Landry et al., 2019). This resulted in negative-to-positive and negative-to-equivocal prediction changes over the time period of this investigation. With the larger training set, the new model provides fewer out-of-domain results and thus changes calls from out-of-domain to valid predictions. The effect of these changes is seen most dramatically in Table 5A but less so in Fig. 5B.

#### 3.2.2. MC models

Since the date of the first official release, CASE Ultra (CU) underwent several major algorithm updates that involved the new methodologies of building and handling statistical and rule-based models. The algorithm to build models was updated with an emphasis on more efficient machine learning and increase predictivity for new test chemicals. The critical 2014 update involved introducing the rule-based model and a built-in consolidation feature, facilitating ICH M7 expert review.

The statistical models were updated during that period as well. The model introduced in 2012 with the training set of 3535 records was rebuilt in 2014 and the bacterial mutagenicity status of each learning set chemical was reevaluated, and several hundred new chemicals were added (Kruhlak, 2013). Another major update was done in 2018, resulting in a new composite bacterial mutagenicity model that was constructed using a training set of 13,514 chemicals with the bacterial mutagenicity data on either G-C or A-T type of mutations (Landry et al., 2019). The additional chemicals introduced new classes of chemicals and improved representation of chemical classes present in the initial versions of the models. This resulted in the identification of new alerts and modulators and overall better coverage of chemical space.

GT_EXPERT is a rule-based model of bacterial mutagenicity. The original rule-based model was introduced in 2014 with 125 alerts; later it was updated with new alerts, bringing the total number to 174 alerts. Currently, it consists of 198 structural alerts, of which 60 represent general mechanisms and the remainder accompanies them as refining factors. These expert alerts were collected from published reviews and scientific studies (Ashby and Tennant, 1988; Bailey et al., 2005; Kazius et al., 2005; Benigni and Bossa, 2008) and were refined and benchmarked by in-house experts on a reference set of 13,514 chemicals with known bacterial mutagenicity outcomes (Sedykh et al., 2015). The increase of the benchmarking set from the original 8556 chemicals to 11, 461 chemicals and to the current 13,514 chemicals not only supported the addition of new alerts but expanded the chemical space covered by the rule-based system. The most significant additional alerts are sulfonic acid halides, acyl halides, sultones, and cyclic sulfates, oxetane, thietane, and azetidine derivatives and aryl hydroxamates. All alerts were also verified using proprietary chemicals (that are not included in the 13,514 chemicals) to ensure performance on pharmaceutical chemicals.

#### 3.2.3. LH models

Derek Nexus is now a relatively mature expert system for the prediction of Ames mutagenicity; however, the knowledge base of this system has continued to develop over the years as new data have become available (both public and private) and new methods explored. A significant change in how Derek Nexus treats compounds which do not activate an alert was made in version 4.0.6 of the program when the concept of negative predictions was introduced (Williams et al., 2016). The approach uses the Dataset Lhasa Ames test reference set in order to determine the confidence with which a negative prediction could be made and if there were any features in the molecule which should be further investigated as there may still be a cause for concern (misclassified features) or were not represented in the Dataset Lhasa Ames test reference set (unclassified). Since the compounds containing unclassified features have been designated as ‘out-of-domain’ (OOD) for the benefit of the current work, this is the reason for the relatively large increase in negative to OOD for this system between the versions indicated in Fig. 5D (red bar). The changes from negative to positive predictions by Derek Nexus are a consequence of the fact that the knowledge base for bacterial mutagenicity expanded from 88 alerts in version 2.0.2 of the software to 133 alerts in version 6.0.1. Of particular note is the fact that a new alert for boronic acids was introduced in Derek Nexus version 3.0.1, following the publication of data outlining the mutagenicity of this compound class (O’Donovan et al., 2011). The introduction of this alert is one of the major reasons for the changes from negative to positive predictions for the different versions of this system shown in Fig. 5A (blue bar). Another reason for a significant number of the changes from a negative to positive prediction was the development of new structural alerts during this time based on Lhasa member donated data. These data are often proprietary and cannot be published directly, however, knowledge can still be gained from it and encoded in an expert rule-based system as it becomes available.

Due to Sarah Nexus being relatively new software, changes to both the method used to build the model and the training set data were made between the early versions of the software as the strengths and limitations of the approach became apparent. The chemical fragmentation method employed in Sarah Nexus was updated between versions 1.2 and 2.0 of the program so that information relating to the chemical environment of different atoms could be retained in the fragments formed from each structure. This change enabled the production of fragments, and therefore hypotheses and predictions, which were more specific than the fragments employed in the previous version. The change led, not only to making existing hypotheses more specific but also the discovery of new, more specific, positive hypotheses. A number of the changes in predictions from positive to negative between these versions, highlighted in Fig. 5B, were the result of the changes to produce these more specific fragments. Changes to training data used to build the model in Sarah Nexus have been made as new data becomes available and more existing data are captured in the sources used to train the model. Lhasa’s Vitic database (Vitic, 2020) is continuously being updated with Ames mutagenicity data extracted from both the public literature and studies donated by Lhasa member organizations. The Sarah Nexus training set inherits new data from this database as well as new data donated directly by members for the improvement of the predictive system. As a result, the Sarah Nexus training set increased from 8201 compounds in version 1.2 to 9882 in version 3.0.0.

### 2.3. Applying computational models

To enable a direct comparison of predictions across software models, the prediction terminology was harmonized; that is, software vendor-specific prediction terminology was mapped to a common terminology (Table 4). Derek Nexus negative predictions with misclassified features were treated as negative for this study. A misclassified feature is a feature in a query molecule which is shared with a non-alerting mutagen in the Lhasa Ames test reference dataset and is not present in any negative compounds in this dataset. In contrast, Derek Nexus negative predictions with unclassified features were treated as out-of-domain (OOD). An unclassified feature is a feature in a query molecule which is not present in any of the structures in the Lhasa Ames test reference dataset (Lhasa, 2020). It is recognized that vendors may recommend a different interpretation of certain output terms, for example Lhasa do not intend “Negative with unclassified features” in Derek Nexus to be interpreted as an OOD result. The OOD term indicates that the compound is not sufficiently (structurally) covered by the model to give a reliable prediction. For the sake of this analysis, a practical approach was taken to harmonize the term in accordance with how many applicants manage the predicted output in practice, and hence such predictions were binned as OODs to match the other software output.

* Structural alerts in Derek Nexus have different levels of confidence associated with them and these take the form of reasoning levels (Judson et al., 2012). An alert with a reasoning level of equivocal indicates that a toxicophore has been identified by the alert writer but there may be reasons why results in the Ames test for the class may not be consistent, or evidence is limited.

### 2.4. Test set evaluation

The final PubChem test set of 3367 chemical structures was evaluated in all current and historical software model versions listed above from each of the three software vendors. For each model and version, the results were tabulated according to the mapped terminology (i.e. the number of positive, negative, equivocal and out-of-domain results were tabulated). The percentage of predictions that changed for the chemicals over time was then evaluated by comparing the initial prediction generated by a software model (e.g. MultiCASE statistical model version A7B) to the most current prediction generated by the contemporary model (e.g. MultiCASE statistical model version GT1-BMUT). Results were then summarized such that the percentage of predictions that changed to a particular result type could be understood (e.g. what percentage of chemicals with predictions that were initially negative, equivocal or out-of-domain generated a positive prediction with the most contemporary version of the software). Results were summarized in this manner for each individual software model included in this investigation. In addition, the percentage of predictions that changed over time was further evaluated taking into consideration consensus predictions. In the context of this investigation, consensus prediction is a prediction that takes into consideration the output of two complementary (Q)SAR models (a statistical model and an expert rule-based model). Consensus predictions were defined as follows:

- A positive prediction in at least one of two models = positive
- An equivocal prediction in at least one model and negative or OOD result in the second model = equivocal
- Negative predictions in both models = Negative
- A negative prediction in one model and OOD result in the second model = OOD
- OOD predictions in both models = OOD

The change in consensus predictions was evaluated by considering the outputs of two complementary systems from a single vendor (intra-vendor consensus predictions) as well as all possible combinations of two complementary systems from two vendors (inter-vendor consensus predictions).

## 3. Results

Table 5 A–C shows the distribution of compounds predicted in each result category (i.e. positive, negative, equivocal, OOD) for each vendor and model version. The associated changes shown as percentages, are tabulated in the supplementary information. It is noted that both LS and MC rule-based models tended to predict fewer compounds as positive as refinement of alerts progressed, decreasing from 1031 compounds in 2014 to 715 in 2018 for LS and similarly, 1014 (2014) to 875 (2018) for MC. In contrast, LH’s rule-based system stayed constant at around 940–960 over the years 2012–2018. The decrease in positive predictions from the LS and MC rule-based systems is accompanied by an increased number of negative predictions, from 2216 to 2314 for LS and from 2134 to 2279 for MC, as well as an increased number of equivocal predictions. This is most markedly true for LS, where the number of equivocal predictions increased from 41 in 2014 to 283 in 2018. For the LH rule-based system, the distribution of negative predictions changed in 2014 when the new categories “Negative with unclassified features” and “Negative with misclassified features” were introduced, and some of the existing negative predictions then fell into one of those new categories.

The statistical model results fluctuated for LS with no specific trend observed. The MC model results showed an increase in negative predictions, 1888 (2012) to 2071 (2018) which was accompanied by a slight decrease in positive predictions and again, a more marked increase in equivocal predictions. The statistical model from LH exhibited the opposite trend with an increase in positive predictions from 593 (2014) to 696 (2018), as well as an increase in negative predictions, 2058 (2014) to 2103 (2018). This was the only model where the number of equivocal predictions decreased over time. It is noteworthy that all models, rule-based and statistical, showed a decrease in number of OOD results over time and that the proportion of OOD results in the set is very small. This highlights that the coverage of the models is high and is improving over time. It is reasonable to assume that this is likely primarily due to an increase in size and diversity of the available training sets.

As the ICH M7 guideline specifies the use of one rule-based and one statistical model, we investigated how many compounds would have a changed consensus call over time, mimicking practical application of the software. The percentage of change in individual model predictions and consensus predictions for each vendor for models released during the time period 2014 to 2018 are shown in Fig. 5A–D. This time interval was selected based upon when the ICH M7 guideline was introduced and the impurity evaluation process became formalized. The introduction of the ICH M7 guideline triggered the development of additional models by software vendors to enable their customers to use both model types from a single vendor, if desired. Prior to this, LS and MC were the primary providers of statistical models and LH the provider of a rule-based model for bacterial mutagenicity predictions.

Of high importance are the cumulative changes in predictions over time from negative or OOD to positive (Fig. 5A). In a scenario where impurity assessments are not reevaluated with every software update, it is of importance to know how likely it is that true positive predictions would be overlooked if not reevaluated, as these could pose a previously unknown safety risk to patients. For individual models, the percentage of compounds with predictions which changed from negative to positive ranged from 0.5% to 4.8% of the compounds. The percentage of predictions which changed from equivocal to positive was 0.1%–4.7% and the smallest change was observed for OOD results which changed to positive, 0.1%–0.9%. For the intra-vendor consensus models, the upper boundary was slightly lower with negative to positive prediction changes ranging 1.4%–2.5%, equivocal to positive prediction changes ranging 1.8%–2.1% and OOD to positive prediction changes ranging 0.1%–0.9%. Changes to equivocal are also of importance if the compounds were previously predicted to be negative or OOD (Fig. 5C). The percentage change from negative to equivocal ranged from 0.03 to 7.3% and for OOD to equivocal, the change was 0.03–0.7%. Conversely, the change from positive to equivocal ranged from 1 to 8.2%.

In comparison, the percentage of compounds where individual model predictions changed from positive to negative (Fig. 5B) ranged from 1.7% to 9.7% and equivocal to negative predictions spanned 0.1%–7.8%. The percentage of compounds where individual predictions changed from OOD to negative (0.6%–4%), was higher compared to percentage of compounds changing from OOD to positive predictions, indicating that an increase in chemical space coverage by models over time tended to include predominately additional negative experimental data. Lastly, changes leading to OOD results (Fig. 5D) were very small and ranged from 0 to 3%, with the 3% associated with changes from negative to OOD by the expert rule-based system from Lhasa. This slightly higher change is the result of an introduction of a new classification category, indicating some features are now flagged as unclassified by the model and which the authors have interpreted as OOD even is this is not a term used by the vendor.

**Fig. 6.**
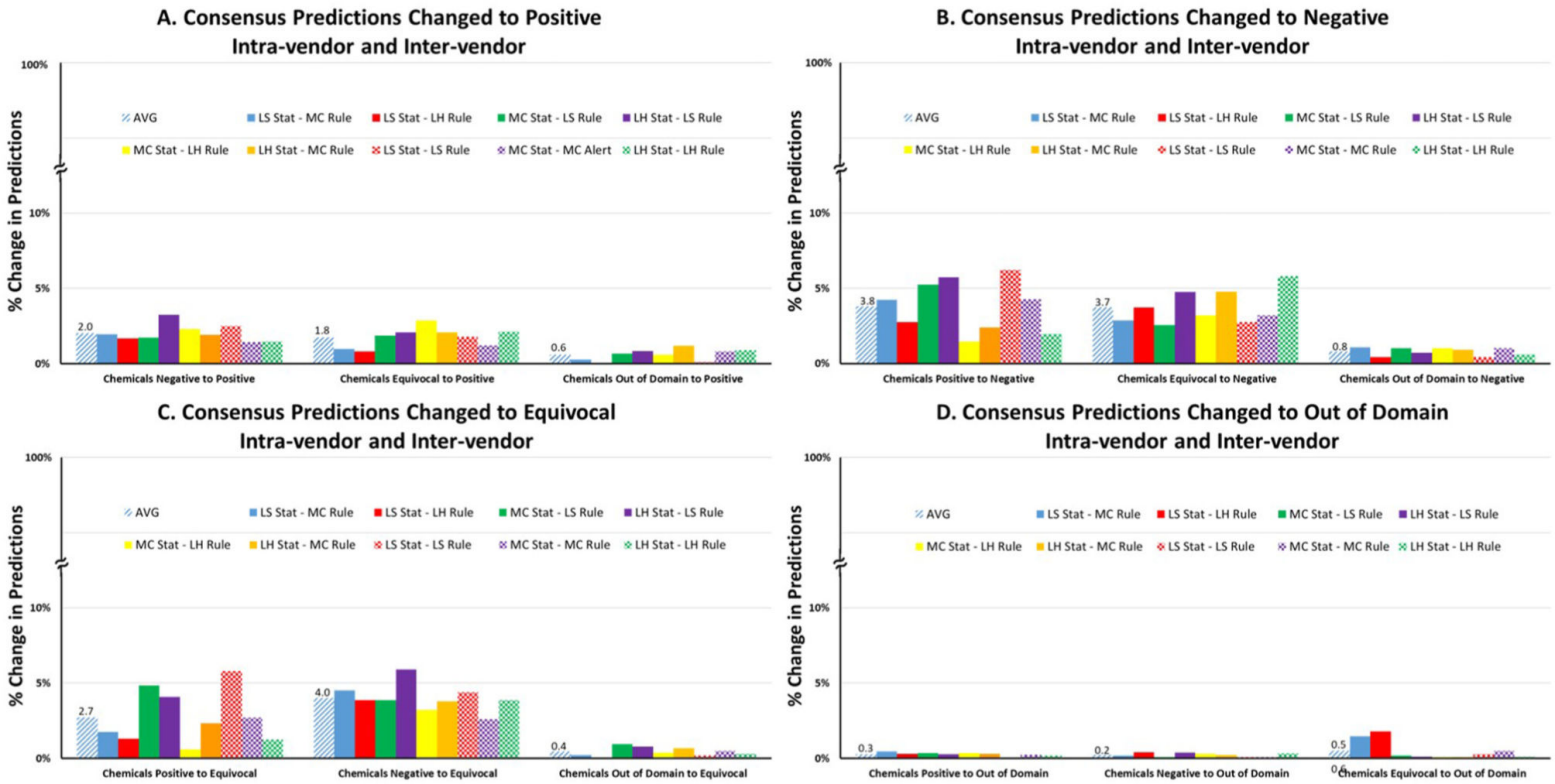
**A-D** Consensus changes for each prediction category.

As pharmaceutical applicants use combinations of models from different vendors, depending on organizational preferences, we analyzed whether these different combinations would result in more or less variation. Fig. 6A–D shows all the classification results from all the inter- and intra-vendor combinations and the results from each pair of models is shown. As illustrated in the figure, there is no significant difference in variation, depending on which combination is used. Intra-vendor variation was very similar to inter-vendor variation with the average prediction change from negative to positive being 2%, equivocal to positive change being 1.8% and the OOD being 0.6% (all illustrated in Fig. 6A). The average changes to negative predictions (Fig. 6B) were slightly higher with positive to negative being 3.8%, equivocal to negative 3.7% and OOD to negative 0.8%. The variation between different consensus combinations for positive and equivocal to negative changes (Fig. 6B) were slightly higher than for the consensus outputs changes to positive. Consensus changes from positive to equivocal (Fig. 6C) were interesting with a more pronounced variation that was dependent on the model combination; ranging from 0.6% to 4.8% (with the largest change being for the LS rule-based and LS statistical combination). Negative to equivocal prediction changes were on average 4% and from OOD to equivocal were 0.4% (both illustrated in Fig. 6C). The smallest changes in consensus predictions, not surprisingly, were associated with changes to OOD (Fig. 6D) and these ranged from 0.2% to 0.5%.

**Fig. 7.**
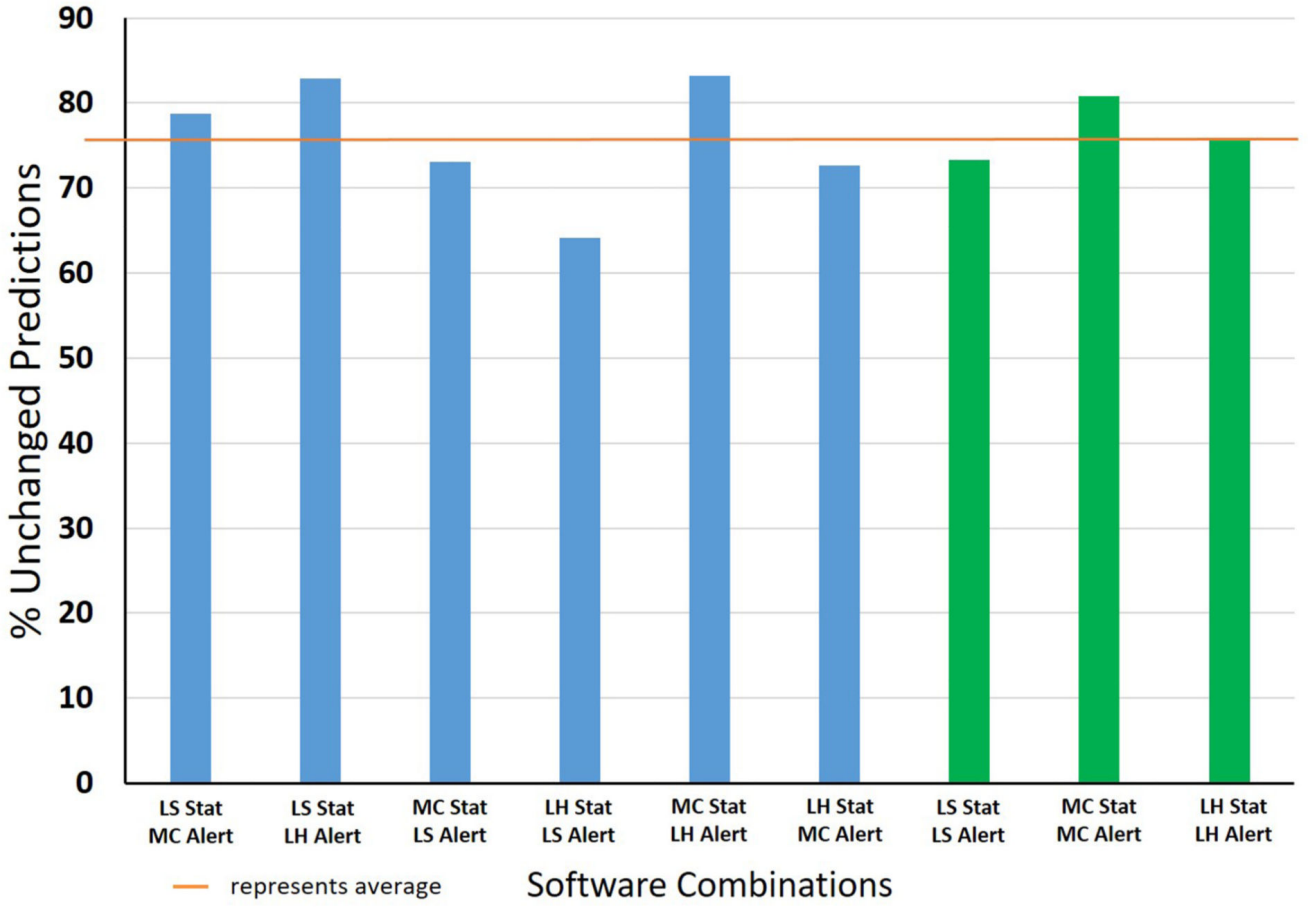
Percentage of chemicals with unchanged predictions of models released between 2014 and 2018. (Blue bars represent inter-vendor combinations and green bars represent intra-vendor combinations, Stat = Statistical model, Alert = Rule based model). Average calculated as the mean.

Taking all possible software combination prediction outputs into account, Fig. 7 shows the percentage of compounds with unchanged consensus predictions for models released between the time period of 2014–2018. The combinations of models with least change associated are the LS or MC statistical model combined with LH rule-based model, followed by MC statistical plus MC rule-based, where all three combinations give over 80% unchanged predictions. Most of the other combinations have over 70% unchanged predictions with the exception of LS rule-based and LH statistical. These are younger systems, both introduced in 2014, that underwent significant refinement between 2014 and 2018. An important finding of this analysis is that the number of unchanged predictions, when complementary models are used in accordance with ICH M7, is very high.

## 4. Discussion

The use of two complementary (Q)SAR models for assessing mutagenic potential of pharmaceutical synthesis impurities is recommended in the ICH M7 guidance. The guidance document also states that models should follow the OECD principles on model validation (OECD, 2007). There are, however, no details on model application, such as how to interpret the model output, leading to a number of “best practice” publications discussing case studies and providing guidance on how to perform expert review (Powley, 2015; Amberg et al., 2016, 2019a; Williams et al., 2016). Similarly, the ICH M7 guidance does not address how to manage software updates or if *in silico* impurity assessments require updates throughout the course of drug development. Consequently, industry practice varies considerably in this respect. As stated earlier, the aim of this study was not to look at model predictivity or overall accuracy in terms of how well the predicted output correlates with experimental results. The goal was to understand the variability in prediction outputs over time, resulting from software updates, and to provide recommendations on how to manage this situation more consistently. As with any analysis of prediction outputs, the results depend partially on the dataset selected for analysis and may not be generally applicable in the global chemical space context. We therefore selected a set of compounds that are as representative as possible of the chemical space covered by pharmaceutical impurities, as this is the use case we were interested in evaluating.

### 4.1. Results from the study

This study considered individual model changes resulting from yearly updates over a time period of 4–8 years, depending on how long each model has been commercially available. In addition, the impact of four years of model updates was examined when models were used in a consensus fashion, consistent ICH M7 guideline recommendations. As outlined in the Results section, the largest changes in consensus predictions are associated with changes from positive or equivocal to negative predictions and from negative to equivocal. Changes from positive to equivocal are in a similar range, but only for some model combinations. From a patient safety point of view, it is reassuring to see that the majority of newer predictions tend to de-risk, rather than identify new risks as models are updated over time. In the same context, changes resulting in equivocal predictions are subject to expert review, which ensures that true positives are identified. It is also interesting to note that the use of two complementary models reduces the variation over time, suggesting that it is not necessary to reevaluate chemicals every time a software update is released. As expected, very few consensus predictions change into OOD results. A prediction that changes to OOD indicates that chemicals have been removed from the training set. This would only happen if the Ames data associated with those chemicals were considered unreliable. Instead, as would be expected when models mature and their included training sets grow and experimental results become more reliable, the number of OOD predictions decreases for all prediction categories, single model or consensus.

### 4.2. Reasons for changes in predictions

There are a number of reasons why a prediction may change between different versions of the same software. The majority of these relate to changes which are designed to lead to an improvement in predictivity of the model and may be a consequence of an improvement in the method used to make the prediction, the encoding of new knowledge or a better curation or reinterpretation of training set data. Examples of each are outlined below.

#### 4.2.1. Updates to modeling methodology

Improving the descriptors and/or algorithms used to make a prediction may lead to more sensitive or specific predictions and therefore making changes to these variables is an active area of research for many software developers. Such improvements may lead to prediction changes between software versions.

The majority of statistical models used in the prediction of bacterial mutagenicity employ descriptors representing fragments of the chemical structures in the training set and associate these sub-structures with either activity or inactivity in the Ames test (Ames et al., 1973a, 1973b; Gee et al., 1994). The method by which these fragments are derived and the structural information they encode may be altered in order to improve predictions. In addition, other descriptors relating to physicochemical properties may be employed (for example molecular weight, ClogP) in order to make the predictions more specific and these may be changed between software versions due to changes in algorithms or training sets used to calculate those descriptors. An example of how an update to the descriptors used in making a prediction may lead to a change in predictions is exemplified in modifications made between Lhasa Limited’s statistical model Sarah Nexus version 1.2 (V.B 2014) and version 2.0.1 (V.C 2016) (Table 3.1). The chemical fragmentation method employed in the program was updated between these versions so that more information relating to the chemical environment of different atoms could be retained in the fragments formed from each structure. This change enabled the production of more specific fragments, and therefore influenced the predictions in the new model version.

Making an overall prediction from the selected descriptors also involves employing an algorithm to combine the descriptor knowledge in order to reach a conclusion. As an example, since the date of the first official release in 2012, CASE Ultra has undergone several major algorithm updates which involved new methodologies for building and refining statistical models (Table 2). The changes affected the discovery and validation of the positive alerts and deactivating features as well as the calculations of the final probability of being positive. The algorithmic changes may also result in changes to predictions over time.

#### 4.2.2. New data leading to new knowledge

New data relating to toxicity endpoints (in this case bacterial mutagenicity data) are continuously being generated and, consequentially, the knowledge that can be derived from these data will also change over time.

In expert rule-based predictive systems, new data and knowledge are usually encoded by creating new structure-activity relationships for compound classes, either by generating new alerts or by refining existing alerts as new, reliable positive data are obtained. Negative data may also lead to the refinement of existing structure-activity relationships and more specific structural alerts. Similar changes to predictions will be observed in statistical systems upon the addition of new data to their training sets. New structure-activity relationships may be derived or the weight of evidence may change for an existing structural feature so that it no longer leads to a positive prediction. Alternatively, the new data may change the similar nearest neighbors to a query compound and therefore the local weight of evidence may change, altering the confidence in a prediction. The introduction of new data may also lead to an increase in the chemical space covered by a model, resulting in a reduction of structures which are outside its applicability domain. In some cases, more confident predictions and a reduction in equivocal results are a consequence of conflicting data being resolved, potentially through experimental data of higher quality becoming available.

An example of new data leading to the generation of a new toxicophore and improving the applicability domain of a model are the studies relating to the bacterial mutagenicity of aryl boronic acids (O’Donovan et al., 2011; Hansen et al., 2015). This work reported a new chemical class of bacterial mutagens which had not previously been known or predicted. Addition of the data, and knowledge associated with it to the *in silico* predictive systems, led to the generation of a new structural alert for prediction of bacterial mutagenicity. The addition of new data for a relatively under-represented chemical class also meant that more chemicals containing this structural feature were brought into the applicability domain of the models.

#### 4.2.3. New interpretation of existing training set data

In addition to new data, existing training set data used to build (Q) SAR models are also continuously being reevaluated and curated. In some cases, earlier model versions contained negative training set chemicals that were only tested in a single Salmonella strain. As model training sets evolved in quality and supporting documentation (often through manual review of studies) some software developers implemented acceptance criteria for training set negatives to be tested in a minimum of two strains (typically TA98 and 100) to minimize the chance that if an additional strain were tested, a positive response would be observed. It is also relevant to ensure that if a compound contains an alert, it has been tested in a strain that is likely to be responsive to that particular structural feature. Other approaches to increasing confidence in limited strain non-mutagenic training set chemicals include only accepting data from a reputable source such as the NTP, or another government agency. While it would be ideal to have all negatives fully tested in all five strains before inclusion in a training set, the reality is that such comprehensive datasets are not available and excluding negatives with limited strain data would lead to substantially lower chemical coverage by the models. In the majority of instances, the reevaluation process results in confirmation of the already existing overall conclusion (i.e. positive or negative). In cases where new conflicting data are introduced, the weight of evidence usually results in the overall conclusion being considered equivocal or unknown. In these cases, the compound is removed from the training set of the model (as are all compounds with overall equivocal calls).

One reason why experimental results may be reevaluated in a model’s training set is when an inadequacy in the particular Ames test protocol employed is revealed that prevents a conclusive assessment of the hazard posed by a compound or chemical class. An example of this is the re-evaluation of the Ames test results of acid halides. It was recently suggested that interaction of acid halides with the test vehicle can potentially confound the evaluation of their mutagenic potential by the Ames test (Amberg et al., 2015). When tested using dimethyl sulfoxide (DMSO) as the vehicle, the compounds can react to produce a reactive halodimethylsulfide species, which may lead to positive results being observed in the Ames test. A non-reactive solvent, such as acetonitrile, is therefore suggested to most accurately measure the bacterial mutagenicity of this compound class, and for most of the acid halides studied in this work, testing in this solvent resulted in a negative response (Amberg et al., 2015).

#### 4.2.4. Factors impacting the effects of software updates

The impact of changes in software versions may not necessarily result in a change in the final classification of an impurity. As discussed earlier, variation in an individual model output may not modify a consensus prediction. For example, a statistical model update resulting in a prediction change from negative to positive may not influence the consensus call, if the rule-based model had already predicted the chemical to be positive. The use of two complementary models introduces a higher sensitivity and ensures that fewer true positives are incorrectly classified. Another important aspect is the use of expert review. Although expert review is stated as “if warranted” in the ICH M7 guideline, it is a common and highly recommended practice to perform expert review to maintain high sensitivity and high negative predictivity, while reducing false positives. Expert review may involve reviewing supporting training set examples, mitigating features, experimental data quality, prediction probabilities of being positive and other factors. Inconclusive predictions, equivocal predictions and OOD results are especially important to review. In these situations, it is common to err on the side of caution and if no clear negative conclusion can be made, and the impurity is often controlled to TTC levels or tested in an Ames test. It may also be prudent to use an additional (Q)SAR model to see if more information is available before resorting to testing.

As mentioned in the introduction, the process of reviewing impurities of the synthetic routes during drug development, is an evolving process. It is common to test some impurities or starting materials early on, especially if the chemical space is novel. The results can often be used as part of an expert review of other impurities and results in substantial knowledge being known about the synthesis products and impurities, by the time of regulatory filing. This internal knowledge also means that changes in predictions will have less practical effect on impurity classifications.

### 4.3. FDA/CDER experience

(Q)SAR models developed for use under ICH M7 are generally updated every one to three years. Updating allows new information to be added to the training sets, errors to be corrected, and improvements to be made to the prediction algorithms and knowledge used to build the models. It is preferred by FDA regulators that pharmaceutical developers re-run (Q)SAR predictions prior to a regulatory filing to ensure that the most current information is provided for review. Despite this, many instances occur where submitted predictions are no longer based upon the most current version of the software by the time they are reviewed, in part due to the timing of new software releases or the need for multiple review cycles.

While it is preferred that applicants use the most current model version, predictions based on older versions are acceptable on a case-by-case basis, particularly within a two-year timeframe. FDA/CDER uses an internal guide for reviewers of 2 years as the “shelf-life” of a (Q)SAR prediction based on experience using the models internally for impurity assessments. This is intended to minimize the burden on pharmaceutical applicants and to reflect FDA/CDER’s experience that generally, when expert knowledge is applied, the predictions do not change substantially within such a timeframe. Also, multiple versions of the same software may be used by applicants for different impurities within a regulatory submission. Since some models are updated on an annual basis, it is recognized that re-running and evaluating a large number of impurities just to add one more impurity prediction at a later time is impractical and unlikely to affect the overall conclusions, especially if expert knowledge is applied to the predictions. Predictions submitted by an applicant are not routinely re-run by FDA/CDER. Furthermore, once a prediction is accepted, an applicant is not expected to update the prediction later in the development program unless a specific cause for concern is identified.

Substantially older predictions, such as those generated prior to ICH M7’s finalization in 2014, are generally unacceptable. When requested by reviewers, FDA/CDER’s computational toxicology group generates predictions with the newest software version to confirm the conclusions based on current knowledge. Additionally, FDA/CDER applies expert knowledge to all internally generated (Q)SAR predictions to serve as a buffer to changes from individual models and increase the likelihood of a high confidence clear positive or negative prediction. For example, in a case where one model generates a negative prediction and the other an equivocal prediction, expert knowledge may be used to confirm that the equivocal result is based on a weak structural alert that is derived from chemicals containing that alert in a different chemical environment than the impurity. Expert knowledge would, in this case, downgrade the equivocal prediction to negative and assign the impurity to Class 5. When the same chemical is predicted at a later date with the next version of the model, the results may be that both models now give negative predictions, directly supporting a Class 5 assignment. The overall conclusion (Class 5) has not changed but the individual predictions have.

To ensure consistency and efficiency in the review of drug applications, FDA/CDER has also implemented the practice of chemical registration to track impurity structures and in-house (Q)SAR assessments across multiple applications. FDA/CDER’s computational toxicology group has established a structure-searchable database where impurity structures are stored along with the results of an internally generated ICH M7 (Q)SAR assessment report containing both prediction results and expert review-based conclusions. When an in-house (Q)SAR assessment is needed for a new impurity, the reviewer can first search the database of previously evaluated structures and retrieve the previously-generated (Q)SAR report, if available. If the report was generated within the last two years, the conclusions can be used directly from the report without the need for a repeat request, thereby reducing duplication of effort for the same structure and ensuring consistency of conclusions across multiple reviews.

## 5. Conclusion

In conclusion, there was a small degree of variability for (Q)SAR Ames statistical and expert rule-based model predictions. The variation is reflective of the updates to the data in the system, new knowledge, and new application of algorithms and is further reduced by the practice of applying two complementary models in a consensus fashion. Most of the variation resulted in positive or equivocal predictions changing to equivocal or negative, respectively, with very few examples (on average 2%) of negative predictions changing to positive predictions.

As a result of this analysis, we conclude that it is unnecessary to re-run a (Q)SAR prediction every time there is a version update unless there are specific reasons to do so, e.g., such as those presented below, which can be determined on a case-by-case basis. These version updates are fairly frequent (often yearly) and updating the predictions can be challenging to manage with no apparent overall value to patient risk. However, we recommend when finalizing the commercial synthetic manufacturing route to consider re-evaluating the impurity assessments, which is in line with the recent ICH M7 (R2) Q&A document (ICH, 2020). This could mean performing (Q)SAR on all actual and potential impurities in the current route or evaluating assessments of individual compounds only. Reviewing software release notes usually provides detailed information on what alerts/chemical classes are affected in an updated model, and this information could be used to highlight which compounds to reassess. It is an organizational choice of which approach is most suitable. Often, considerable knowledge about the synthetic route has also been gained over the development process through experimental Ames testing and it is reasonable to assume that with a diligent process over the course of development, very few surprises would happen at time of commercialization. This knowledge, together with a final review, using contemporary software models, will ensure patient safety and that the best scientific approach is applied.

## Supplementary Material

1

2

## Figures and Tables

**Table 1:** Leadscope models.

Year	Leadscope version	Statistical-based QSAR	Rule-based SAR	Type of update
2010	LSE 2.7	Salmonella v2 (3579 compounds)	N/a	statistical model features updated
2013	LSE 3.1	Salmonella v3 (3979 compounds)	N/a	statistical model training set and features updated
2014	LSE 3.2	Salmonella v3 (3979 compounds)	Genetox Alerts v1 (162 rules)	first alerts release
2015	LSE 3.3	Salmonella v3 (3979 compounds)	Genetox Alerts v2 (215 rules)	alerts updated
2016	LSE 3.4	Salmonella v3 (3979 compounds)	Genetox Alerts v3 (213 rules)	alerts updated
2017	LSE 3.5	Salmonella v3 (3979 compounds)	Genetox Alerts v4 (224 rules)	alerts updated
2018	LSE 3.6	Bacterial Mutation v1 (9189 compounds)	Genetox Alerts v5 (226 rules)	alerts updated, statistical model training set and features updated

**Table 2:** MultiCASE models.

Year	CASE Ultra version	Statistical-based QSAR	Rule-based SAR	Type of update
2012	1450	A7B (3535 records)	N/a	First official release
2013	1460	A7B (3535 records)	N/a	Minor update
2014	1520	GT1_A7B (3979 records)	GT_EXPERT (125 rules, 8556 records)	Major algorithm update, new models
2015	1603	GT1_A7B (3979 records)	GT_EXPERT (125 rules, 8556 records)	Major algorithm update
2017	1623	GT1_A7B (3979 records)	GT_EXPERT (174 rules, 11461 records)	Minor update
2018	1704	GT1_BMUT (13514 records)	GT_EXPERT (198 alerts, 13514 records)	Major algorithm and models update

**Table 3:** Lhasa models.

Year	Nexus version	Statistical-based QSAR	Rule-based SAR	Type of update
2012	1.5	n/a	2.0.2	Updates to Derek Nexus knowledge base.
2013	1.5	n/a	3.0.1	Updates to Derek Nexus knowledge base, including introduction of alert for aryl boronic acids.
2014	1.7.6	1.1.2	4.0.6	Sarah Nexus new software. Updates to Derek Nexus knowledge base. Introduction of negative predictions in Derek Nexus.
2014	2.0	1.2	4.1	Updates to Derek Nexus knowledge base. Changes to Sarah Nexus software interface.
2016	2.1.1	2.0.1	5.0.2	Update to Sarah Nexus fragmentation algorithm. Updates to Sarah Nexus training set. Updates to Derek Nexus knowledge base.
2018	2.2.1	3.0.0	6.0.1	Updates to Sarah Nexus training set. Updates to Derek Nexus knowledge base.

**Table 4:** Mapping model specific output to common analysis terms.

Mapped Term	Leadscope Statistical	Leadscope Rule-Based	MultiCASE Statistical	MultiCASE Rule-Based	Lhasa Statistical	Lhasa Rule-Based
**Positive**	Positive	Positive	Positive	Positive	Positive	Alerting Structure with reasoning of equivocal or higher*
**Negative**	Negative	Negative	Negative	Negative	Negative	Negative/Negative with misclassified features
**Equivocal**	Indeterminate	Indeterminate	Inconclusive	Inconclusive	Equivocal	–
**Out-of-Domain (OOD)**	Not in domain	Not in domain	Out of domain	Out of domain	Outside domain	Negative with unclassified features

**Table 5 A:** Number of compounds predicted in each category of the Leadscope models.

General Metric Descriptor	Leadscope Statistical			Leadscope Rule Based					Leadscope Consensus				
	V2 (2010)	V3 (2013)	V4 (2018)	V1 (2014)	V2 (2015)	V3 (2016)	V4 (2017)	V5 (2018)	V1 (2014)	V2 (2015)	V3 (2016)	V4 (2017)	V5 (2018)
# Chemicals Positive	502	505	666	1031	1000	927	718	715	1811	1164	1095	962	923
# Chemicals Negative	2312	2413	2225	2216	2222	2264	2246	2314	1894	1891	1930	1911	1975
# Chemicals Equivocals	308	287	407	41	89	119	347	283	213	256	285	438	400
# Chemicals out of domain	245	162	69	79	56	57	56	55	79	56	57	56	69

**Table 5 B:** Number of compounds predicted in each category of the MultiCASE models.

General Metric Descriptor	MultiCASE Statistical						MultiCASE Rule Base				MultiCASE Consensus			
	V1450 (2012)	V1460 (2013)	V1520 (2014)	V1603 (2015)	V1623 (2017)	V1705 (2018)	V1520 (2014)	V1603 (2015)	V1623 (2017)	V1704 (2018	V1520 (2014)	V1603 (2015)	V1623 (2017)	V1704 (2018)
# Chemicals Positive	985	985	683	683	683	654	1014	954	954	875	1188	1139	1139	1061
# Chemicals Negative	1888	1888	1961	1963	1962	2071	2134	2175	2175	2279	1690	1733	1732	1839
# Chemicals Equivocals	319	319	530	530	530	496	16	49	49	60	249	262	496	294
# Chemicals out of domain	175	175	193	191	192	145	201	187	187	153	239	232	233	173

**Table 5 C:** Number of compounds predicted in each category of the Lhasa models.

General Metric Descriptor	Lhasa Statistical			Lhasa Rule Based						Lhasa Consensus		
	V.B (2014)^a^	V.C (2016)	V.D (2018)	V.A (2012)	V.B (2013)	V.C (2014)	V.D (2014)	V.E (2016)	V.F (2018)	V.A (2014)	V.B (2016)	V.C (2018)
# Chemicals Positive	593	741	696	1006	1004	1009	1009	997	1009	1183	1265	1220
# Chemicals Negative	2058	2029	2103	2361	2363	2256	2256	2274	2255	1666	1698	1759
# Chemicals Equivocals	553	461	441							346	267	256
# Chemicals out of domain	163	136	127	0	0	102	102	96	103	172	137	132

a Version A and B of the Lhasa statistical model produce the same results for the dataset. This is a consequence of the fact that changes between these versions were made only to the software interface rather than the model itself.
